# VIEWPOINT FROM THE DIVISION OF NEUROSCIENCE

**DOI:** 10.1093/geroni/igz038.1640

**Published:** 2019-11-08

**Authors:** Eliezer Masliah

**Affiliations:** National Institute on Aging, National Institutes of Health, Bethesda, Maryland, United States

## Abstract

Dr. Masliah will review accomplishments of the Division of Neurosciences and make projections for the future.

